# Nature’s hidden gem: quercitrin’s promising role in preventing prostate and bladder cancer

**DOI:** 10.2144/fsoa-2023-0041

**Published:** 2023-05-16

**Authors:** Benito Fabio Mirto, Luca Scafuri, Enrico Sicignano, Ciro De Luca, Pasquale Angellotto, Giuseppe Di Lorenzo, Daniela Terracciano, Carlo Buonerba, Alfonso Falcone

**Affiliations:** 1 Department of Neurosciences, Reproductive Sciences & Odontostomatology, Federico II University, Naples, Italy; 2 Oncology Unit, Hospital ‘Andrea Tortora’, ASL Salerno, Pagani, Italy; 3 Associazione O.R.A., Somma Vesuviana, Naples, Italy; 4 Department of Medicine & Health Science, University of Molise, Campobasso, Italy; 5 Department of Translational Medical Sciences, University ‘Federico II’, Naples, Italy

**Keywords:** antineoplastic activity, antioxidants, bladder cancer, fisetin, flavonoids, kaempferol, myricetin, preventive agent, prostate cancer, quercitrin

Flavonols are a subgroup of flavonoids, a diverse class of natural compounds found abundantly in the plant kingdom [1]. The basic chemical structure of flavonols comprises a benzene ring and a pyran ring joined by a carbon bridge, with the B ring being the site of hydroxylation and glycosylation [1]. Flavonols have been reported to possess various health-promoting properties, including antioxidant, anti-inflammatory and anticancer effects, as well as the ability to modulate cellular signaling pathways [1]. Quercetin [2] is a flavonol that is naturally present in several fruits, vegetables and grains, including apples, onions and berries. It possesses potent antioxidant and anti-inflammatory properties, which have been shown to provide a range of health benefits. In addition to its antineoplastic activity against prostate and bladder cancer, quercetin has been found to have positive effects on cardiovascular health, reducing the risk of heart disease and stroke. It also has neuroprotective properties and may improve cognitive function. Furthermore, quercetin has been shown to have anti-allergic and antiviral effects, potentially reducing the severity of allergic reactions and viral infections. Unlike quercetin, which is an aglycone, quercitrin is bound to a sugar molecule, which enhances its bioavailability and absorption in the body. Several *in vitro* and *in vivo* studies have investigated the potential of quercitrin as a preventive and therapeutic agent against a variety of malignancies, including two highly prevalent neoplasms in men such as prostate and bladder cancer [3].

Prostate and bladder cancer represent a major cause of cancer related death in North America and Europe [1,4,5]. Although there have been considerable improvements in diagnostic techniques [5,6], prognostic factors [4,7] and therapeutic options [8,9], preventive measures remain the key to reduce morbidity and mortality associated with prostate and bladder cancer. Due to the impact of dietary factors on the risk of developing both bladder and prostate cancer [10,11], prevention through dietary interventions has gained significant attention in recent years. Given its excellent safety profile, low cost and the extensive evidence supporting its antineoplastic activity, quercitrin appears as an excellent candidate as a preventive agent against both prostate and bladder cancer, both in the general population and in selected individuals at higher risk – for example, those carrying specific genetic mutations, such as BRCA2 gene alteration [12] and smokers [13].

Numerous *in vitro* and animal studies have shown promising results supporting the antineoplastic effect of quercitrin against prostate cancer. Quercetin has been found to induce apoptosis in prostate cancer cell lines LNCaP and PC3 in a dose-dependent manner, with significant apoptosis observed at concentrations of 40 μM and above. In addition, quercetin treatment resulted in decreased expression of the anti-apoptotic protein Bcl-2 and increased expression of the pro-apoptotic protein Bax. *In vivo* studies showed that quercetin treatment significantly reduced tumor volume in mice bearing LNCaP and PC3 tumors [14].

Conversely, there is limited clinical data available on its effectiveness against prostate cancer in humans.

McCann *et al.* [15] found that high intakes of the quercetin were associated with a decreased risk of prostate cancer in a study designed to investigate the relationship between intakes of selected nutrients, foods and phytochemicals and prostate cancer risk in western New York. The study was conducted as a population-based case–control study, with 697 men newly diagnosed with prostate cancer and 666 controls enrolled. The study found that high intakes of vitamin E, selenium and the phytochemical quercetin were associated with a decreased risk of prostate cancer, while high intakes of total fat and saturated fat were associated with an increased risk. The study also found that high intakes of fruits and vegetables were associated with a decreased risk of prostate cancer, while high intakes of dairy products were associated with an increased risk. Specifically, the study found that men with the highest quintile of quercetin intake had a 50% lower risk of prostate cancer compared with men with the lowest quintile of quercetin intake. This association was statistically significant even after adjustment for potential confounding factors such as age, BMI, family history of prostate cancer and total energy intake.

As for bladder neoplasms, a wealth of preclinical and epidemiological evidence supports its potential as a preventive anticancer agent. As an example, one recently published preclinical study showed that treatment with quercetin at 40 and 60 μM resulted in decreased cell viability and cell number in T24 bladder cancer cells. The study also found that quercetin treatment induced morphological changes in T24 bladder cancer cells such as decreased cell body, cytoplasmic retraction and membrane condensation, as well as an increased percentage of nuclei characteristic to the apoptotic and senescence processes [16]. Another study found that glycoside derivate isoquercetin, that is converted *in vivo* into quercetin [17], inhibited cell proliferation and induced apoptosis in human bladder cancer cells by arresting the cell cycle in the G1 phase. This study also demonstrated that isoquercitrin inhibited xenograft tumor growth in nude mice [18]. Epidemiological studies have consistently found an inverse association between quercetin intake and bladder cancer risk. A meta-analysis was conducted to examine the link between dietary flavonoid intake and the risk of smoking related cancers. The analysis included data from 21 observational studies, which revealed that consuming flavonoids, specifically quercetin, was associated with a lower risk of smoking related cancers. The pooled relative risk for high versus low quercetin intake was 0.76 (95% CI: 0.64–0.89), indicating a 24% reduction in the risk of smoking related cancers among individuals with high quercetin intake compared with those with low quercetin intake. Specifically, the study showed that the protective effect of quercetin against smoking related cancer was more pronounced in bladder cancer than in other smoking related cancers. The pooled relative risk for high versus low quercetin intake was 0.62 (95% CI: 0.50–0.76) for bladder cancer, indicating a 38% reduction in the risk of bladder cancer among individuals with high quercetin intake [19].

As previously discussed, the beneficial effects of quercitrin may be shared by other flavonols, including myricetin and kaempferol. In a large cohort study evaluating flavonoid intake in 10,054 individuals based on dietary habits and flavonoid concentrations in Finnish foods, incident cases of various diseases were computed from national public health registers. The total daily intake of myricetin for the entire cohort was 0.12 mg. Of all the malignancies considered, myricetin consumption was only associated with a lower risk of prostate cancer, with a significantly lower risk observed in the fourth and third quartiles compared with the first [1]. In an epidemiological study based on the Netherlands Cohort Study, the potential association between dietary flavonoid intake, black tea consumption and the risk of prostate cancer was evaluated. The study cohort included 58,279 men who provided detailed baseline information on various cancer risk factors, and between 1986 and 2003, 3362 cases of prostate cancer were identified, including 1164 cases of advanced (stage III/IV) cancer. The findings suggest that individuals who consume higher amounts of total catechin, epicatechin, kaempferol and myricetin, as well as those who drink black tea, may have a lower risk of advanced stage prostate cancer. A statistically significant decrease in the hazard ratios for stage III/IV and stage IV prostate cancer was observed in individuals who consumed the highest amount of black tea (≥5 cups/day) compared with those who consumed the lowest amount (≤1 cup/day) [20].

The evidence reviewed here suggests that oral supplementation with quercitrin may be a potential preventive measure against both prostate and bladder cancer in the general population, as well as in individuals with a higher risk. Quercitrin is a natural compound that is present in several fruits and vegetables, including apples, onions and grapes. However, the levels of quercitrin in these foods are relatively low, and obtaining a pure compound of quercitrin for use as a supplement may be difficult. One option to overcome this limitation is to develop a standardized extract of quercitrin from natural sources. This approach could help ensure that the supplement contains a consistent amount of quercitrin and potentially improve the bioavailability of the compound. Another approach to increasing the efficacy of quercitrin supplementation is to combine it with other flavonoids, such as myricetin and kaempferol, which have similar beneficial effects. This combination could potentially enhance the preventive properties of quercitrin against prostate and bladder cancer. In addition to the difficulties associated with obtaining a pure compound of quercitrin, protecting the intellectual property of quercitrin supplementation may also be challenging. As a natural compound, quercitrin cannot be patented, which may make it less attractive for pharmaceutical companies to invest in its development. However, alternative approaches, such as developing standardized extracts or combining quercitrin with other flavonoids, may help overcome these challenges.

In conclusion, the potential preventive properties of quercitrin against prostate and bladder cancer warrant further investigation through clinical research. Clinicians should discuss with their patients the potential benefits and limitations associated with quercitrin supplementation and encourage a balanced and varied diet that includes fruits and vegetables rich in quercitrin and other flavonoids.
